# Report on emerging technologies for translational bioinformatics: a symposium on gene expression profiling for archival tissues

**DOI:** 10.1186/1471-2407-12-124

**Published:** 2012-03-29

**Authors:** Levi Waldron, Peter Simpson, Giovanni Parmigiani, Curtis Huttenhower

**Affiliations:** 1 Department of Biostatistics and Computational Biology, Dana-Farber Cancer Institute, Boston, MA, USA; 2 Department of Biostatistics, Harvard School of Public Health, Boston, MA, USA; 3 The University of Queensland, UQ Centre for Clinical Research, Herston, Brisbane, QLD 4029, Australia

## Abstract

**Background:**

With over 20 million formalin-fixed, paraffin-embedded (FFPE) tissue samples archived each year in the United States alone, archival tissues remain a vast and under-utilized resource in the genomic study of cancer. Technologies have recently been introduced for whole-transcriptome amplification and microarray analysis of degraded mRNA fragments from FFPE samples, and studies of these platforms have only recently begun to enter the published literature.

**Results:**

The Emerging Technologies for Translational Bioinformatics symposium on gene expression profiling for archival tissues featured presentations of two large-scale FFPE expression profiling studies (each involving over 1,000 samples), overviews of several smaller studies, and representatives from three leading companies in the field (Illumina, Affymetrix, and NuGEN). The meeting highlighted challenges in the analysis of expression data from archival tissues and strategies being developed to overcome them. In particular, speakers reported higher rates of clinical sample failure (from 10% to 70%) than are typical for fresh-frozen tissues, as well as more frequent probe failure for individual samples. The symposium program is available at http://www.hsph.harvard.edu/ffpe.

**Conclusions:**

Multiple solutions now exist for whole-genome expression profiling of FFPE tissues, including both microarray- and sequencing-based platforms. Several studies have reported their successful application, but substantial challenges and risks still exist. Symposium speakers presented novel methodology for analysis of FFPE expression data and suggestions for improving data recovery and quality assessment in pre-analytical stages. Research presentations emphasized the need for careful study design, including the use of pilot studies, replication, and randomization of samples among batches, as well as careful attention to data quality control. Regardless of any limitations in quantitave transcriptomics for FFPE tissues, they are often the only biospecimens available for large patient populations with long-term history and clinical follow-up. Current challenges can be expected to remain as RNA sequencing matures, and they will thus motivate ongoing research efforts into noise reduction and identification of robust, translationally relevant biological signals in expression data from FFPE tissues.

## Text

The Emerging Technologies for Translational Bioinformatics symposium on gene expression profiling for archival tissues was held on August 5, 2011, in response to interest from numerous researchers in the planning stages of large-scale expression profiling studies using clinical FFPE tissues. While a number of smaller-scale studies have demonstrated technical feasibility of FFPE expression profiling, it remains a novel technology with great potential but still-unknown risks and challenges. This symposium brought together some of the most experienced researchers in the field, to share experiences and provide an early look at the particular risks and issues involved.

The symposium comprised two keynote presentations by Dr. Jeannette Eckel-Passow and Dr. Mickey Williams, each discussing their experiences with large, published studies featuring FFPE gene expression data. Dr. Eckel-Passow is a statistician for the North Central Cancer Treatment Group-led N9831 intergroup clinical trial at the Mayo Clinic in Minnesota, USA, and analyzed over 1,500 FFPE HER-2 positive breast tumor specimens from 400 centers in the largest FFPE expression profiling study yet undertaken. Dr. Williams is one of the longest-standing researchers in archival tissue gene expression profiling, having performed several of the earliest RT-PCR studies, and he discussed assay development specifically for clinical applications using expression profiling of FFPE tissues. Other presentations featured as-yet-unpublished studies from the University of Queensland (by Dr. Peter Simpson), Harvard School of Public Health (by Dr. Levi Waldron), and Dana-Farber Cancer Institute (by Dr. John Quackenbush). These investigations featured novel methods development for the analysis of FFPE expression profiles, which represent a unique resource but which require additional planning, quality control, and care in interpretation relative to studies of frozen tissues. The symposium provided an early look at the challenges that will become increasingly widespread as expression profiling of FFPE tissues enters the mainstream, and it provided practical guidelines for the planning and analysis of such experiments.

### Planning of large-scale FFPE expression profiling studies

Planners of FFPE expression studies must select an analysis platform in the face of limited and sometimes contradictory pieces of evidence. Considerations in these pre-study stages were discussed by a panel of three investigators currently completing such designs for FFPE profiling, Dr. Lorelei Mucci (Harvard School of Public Health), Dr. Michael Birrer (Massachusetts General Hospital), and Dr. Matthew Freedman (Dana-Farber Cancer Institute). Among the current alternatives available for whole-genome profiling are Illumina's WG-DASL^® ^assay or NuGEN/Affymetrix for amplification/hybridization, or profiling of a limited gene panel by Nanostring nCounter technology. Since the particulars of sample fixation and storage have lasting influence on expression profiling, all panel members undertook targeted pilot projects before moving ahead on a large scale. A central theme in this discussion was the decision whether to optimize accuracy by assaying only a panel of selected candidate genes, or to take a whole-genome approach with potentially lower accuracy. The development of methods for selecting a candidate gene panel was identified as a pressing research need for the bioinformatics and biostatistics communities. The experimental design phase of planning was discussed by Dr. Eckel-Passow, who emphasized attention to randomization, to ensure balance of any variables of interest across the order of RNA extraction and across 96-well PCR plates, as well as the use of positive control samples on each plate, and within-plate and between-plate replication. These steps enabled post-hoc identification of problematic batches which could then be repeated, and ensured that batch-specific variations were not confounded with the outcome of interest.

### Analysis of FFPE expression data

Microarray data from FFPE tissues show overall greater amounts of noise and technical effects than would be expected from fresh-frozen tissues, a message presented by all four morning speakers. In the analysis of expression data from 1,500 clinical trial FFPE specimens, Dr. Eckel-Passow demonstrated how visualization of raw data, through box plots sorted by plate and extraction order, enabled post-hoc identification of issues affecting data quality at different parts of the experiment, so that the affected samples could be removed, re-assayed, and otherwise corrected for in downstream analysis. She presented a novel "stress" metric for quality control, which quantified the extent of changes to raw expression values during normalization and identified samples with unusually compressed or skewed distributions of raw intensity measurements. Dr. Levi Waldron noted an approximately 20% rate of sample failure in a study of 1,003 colorectal cancer specimens from long-term health studies, and showed that strict quality control improved reproducibility in the ranking of differentially expressed genes and of probe-level measurements between replicates. Two other speakers presented smaller-scale Illumina DASL^® ^technical studies, highlighting the risks still present from assay and sample variability, and the need for pilot studies using samples from the actual study population. Dr. Peter Simpson from the University of Queensland, Australia, presented two published breast cancer microarray studies which showed promising results [1,2], but more recently experienced 70% sample failure rate in a 96-sample breast cancer experiment. These sample failures were not predicted from sample age, qPCR of housekeeping genes, or RNA quality or quantity. Dr. Simpson also observed that in technical replicate measurements of the same sample by Illumina WG-DASL^®^, some probes were detected in one replicate and not the other. Dr. Mickey Williams pointed out during subsequent questions that mRNA transcripts are amplified from very low levels for quantitation in FFPE tissues, to the extent that a single intact transcript segment can be measured, which could result in noticeable random variations between technical replicates.

To close the morning, Dr. John Quackenbush of the Dana-Farber Cancer Institute reported on a pilot study of Illumina DASL^® ^for the DRIVE U19 breast cancer project. In this pilot study, sample expression profiles clustered more strongly by RNA input concentration than by Estrogen Receptor status, and the decision was made to move from whole-transcriptome analysis to an 800-gene panel assayed using Nanostring technology. This presentation also provided a glimpse into the future of RNA-seq, which was assessed in a pilot study of seven FFPE bladder cancer samples. The analysis process was similar to RNA-seq for fresh-frozen samples without the need to fragment RNA, using DSN normalization. This process reduces ribosomal RNA abundance, necessary since poly-A selection cannot be performed on FFPE tissues, and the resulting expression profiles showed promising separation of proliferative and non-proliferative tumor types.

### State of the technology, by industry representatives

Industry representatives from Illumina, NuGEN, and Affymetrix summarized their solutions for expression profiling of FFPE samples by microarray and RNA-seq, as well as for methylome profiling.

Illumina provides the WG-DASL^® ^HT-12 v4 system for cDNA labeling, sequence-specific amplification, and hybridization. This system differs from other approaches in that cDNA amplification is limited to oligo-targeted sequences, for specificity of detection. Illumina has recently developed two technologies enabling RNA-seq on FFPE tissues as well, and additionally offers the Infinium HumanMethylation450 BeadChip for whole-genome methylations study of FFPE tissues.

NuGEN discussed their Ovation^® ^FFPE RNA Amplification System v2, which can be used to amplify picogram quantities of starting RNA, using a proprietary SPIA process for linear DNA amplification at constant temperature. The amplified cDNA product can be used in combination with any microarray platform or with RNA-seq, and an example was shown of equivalence in results obtained from RNA-seq and microarray with common NuGEN preparation. This process was recently revised to increase sensitivity, and results were shown that demonstrated increased detection of biologically relevant genes as compared to previous versions.

An Affymetrix representative indicated that their microarrays, including the familiar Human Genome U133 Plus 2 and newer exon arrays, can be employed directly with RNA from FFPE tissues. These are normally used in combination with NuGEN Ovation^® ^sample preparation, and successful examples of such combinations were presented by both Affymetrix and NuGEN. The Affymetrix miRNA array and some arrays used for GWAS and copy number can also be used for FFPE samples.

When asked about future trends in the technology, industry speakers noted a trend towards next-generation sequencing, and toward integrating genetic, epigenetic and expression analyses of FFPE samples.

### "De-Risking" FFPE expression profiling for clinical assay development

In the clinical assay development, it is critically important to avoid mistakes in diagnosis. To this end it may be necessary to allow an outcome of "indeterminate" in some cases. Afternoon keynote speaker Mickey Williams, from the Patient Characterization Center and Clinical Assay Development Center, SAIC-Frederick, Inc., presented a case study in the development of an expression-based diagnostic assay for diffuse large B-cell lymphoma from FFPE tissues, using NuGEN sample preparation and Affymetrix microarrays [3]. Assay development required fresh-frozen tissues to establish a model to sub-classify diffuse large B-cell lymphomas into the prognostic subgroups germinal center B-cell (GCB) and activated B-cell (ABC), which was then extended to FFPE tissue samples. FFPE tissues provided lower-quality data, but remained valuable for accurate disease classification. With the central importance of accuracy in clinical assay development in mind, Dr. Williams outlined steps to "de-risk" expression profiling from FFPE tissues:

• RNA concentration and Bioanalyzer RNA Integrity Number (RIN) were often not sufficiently predictive of true expression data quality.

• qRT-PCR of a housekeeping gene proved very informative and was usable for quality assurance of each sample by defining an acceptable range of Ct scores. Low linear amplification yield was indicative of poor sample quality.

• Ambient moisture during tissue storage or handling can be unavoidable, but was consistently observed to degrade subsequent expression assays.

• Developers of clinical assays should demand an adjacent section for H & E staining, to confirm diagnosis and assess cellularity which may impact gene expression measurements.

## Summary

FFPE tissues represent an irreplaceable and under-utilized library of the trancriptomes of large patient populations with long-term clinical follow-up, and such samples are utilized for virtually all routine pathology tests. However, expression data from such tissues typically include more noise than do data from fresh-frozen tissues, a cost of their closeness to the clinic and widely varied storage and handling processes. FFPE expression data are more prone to sample bias and failure, and they can vary widely even among different samples of the same tissue type. Symposium presenters suggested steps to successfully overcome these challenges, including the use of pilot studies, positive control samples, replication, balanced randomization of samples to technical processes, and awareness of probable batch effects and low-quality samples during data analysis. RNA-seq will likely soon be a viable alternative to microarray hybridization, but sample characteristics introduced by the FFPE preservation process will remain. Numerous FFPE studies of unprecedented scale in the microarray literature are in their planning and execution stages, and the field can expect to continue exploring the analysis and interpretation of these archival tissue gene expression data.

## Abbreviations

Ct: Cycle Threshold; qRT-PCR: Quantitative Real-time PCR; RIN: RNA Integrity Number; FFPE: Formalin-Fixed Paraffin-Embedded; H & E: Hematoxylin and Eosin Stain; DSN: duplex-specific nuclease; WG-DASL: Whole-Genome cDNA-mediated Annealing Selection, extension, and Ligation.

## Competing interests

The authors declare no competing interests. This symposium was supported in part by registration fees from Illumina Inc., NuGEN Inc., and Affymetrix Inc.

## Authors' contributions

LW, GP, and CH organized the symposium. LW drafted the manuscript. PS, GP, and CH contributed to manuscript preparation. All authors read and approved the final manuscript.

## Note

A symposium held on August 5, 2011 at the Dana-Farber Cancer Institute

## Pre-publication history

The pre-publication history for this paper can be accessed here:

http://www.biomedcentral.com/1471-2407/12/124/prepub
